# Supplementary material for: PRImary care Management of lower Urinary tract Symptoms in men: protocol for development and validation of a diagnostic and clinical decision support tool (the PriMUS study)
Source: BMJ Open. 2020 Jun 30;10(6):e037634. doi: 10.1136/bmjopen-2020-037634 (PMC7328815; doi:10.1136/bmjopen-2020-037634)
Supplement: Supplementary data [file bmjopen-2020-037634supp001.pdf]

Screening and Confirmation of Eligibility

The GP will confirm eligibility to enter the study. The test results needed to confirm eligibility are: digital rectal examination, physical examination and prostate specific antigen blood test.

↓

Study Visit (Part A)

- Consent *(If not already taken in GP Visit).*
- Collection/Provision of Bladder Diary.
- Two self-reported questionnaires.
- Questions regarding medical history, relevant medical and demographics.

↓

Study Visit (Part B)

- Urodynamics reference test.
- Uroflowmetry (Flowtaker).

↓

Follow Up Phone Call

The nurse who conducted the urodynamics test will call the patient 3 days after the test to monitor any adverse events.

If the nurse then feels there are no ongoing side effects from the urodynamics, the patient will start using the Flowtaker.

↓

GP Summary Report

Once all the results are available, they will be fed back to the GP. An appointment will be arranged with the patient for further treatment and management of LUTS.

↓

6 Month Note Review

Review of the patient's medical notes will take place 6 months after the patient's treatment and management decision with the GP.
