# Supplementary material for: PRImary care Management of lower Urinary tract Symptoms in men: protocol for development and validation of a diagnostic and clinical decision support tool (the PriMUS study)
Source: BMJ Open. 2020 Jun 30;10(6):e037634. doi: 10.1136/bmjopen-2020-037634 (PMC7328815; doi:10.1136/bmjopen-2020-037634)
Supplement: Author's manuscript [file bmjopen-2020-037634.draft_revisions.pdf]

BMJ Open is committed to open peer review. As part of this commitment we make the peer review history of every article we publish publicly available.

When an article is published we post the peer reviewers' comments and the authors' responses online. We also post the versions of the paper that were used during peer review. These are the versions that the peer review comments apply to.

The versions of the paper that follow are the versions that were submitted during the peer review process. They are not the versions of record or the final published versions. They should not be cited or distributed as the published version of this manuscript.

BMJ Open is an open access journal and the full, final, typeset and author-corrected version of record of the manuscript is available on our site with no access controls, subscription charges or pay-per-view fees (http://bmjopen.bmj.com).

If you have any questions on BMJ Open's open peer review process please email info.bmjopen@bmj.com

BMJ Open

Protocol for PRImary care Management of lower Urinary tract Symptoms in men: Protocol for development and validation of a diagnostic and clinical decision support tool (The PriMUS Study).

Journal:	*BMJ Open*
Manuscript ID	bmjopen-2020-037634
Article Type:	Protocol
Date Submitted by the Author:	11-Feb-2020
Complete List of Authors:	Pell, Bethan; Cardiff University Centre for Trials Research,<br>Thomas-Jones, Emma; Cardiff University, Centre for Trials Research<br>Bray, Alison; Newcastle Upon Tyne Hospitals NHS Foundation Trust, Medical Physics; Newcastle Upon Tyne Hospitals NHS Foundation Trust, Translational and Clinical Research Institute<br>Agarwal, Ridhi; University of Birmingham, Test Evaluation Research Group, Institute of Applied Health Research,<br>Ahmed, Haroon; Cardiff University, Division of Population Medicine<br>Allen, A; Newcastle University, National Institute for Health Research (NIHR), Newcastle In Vitro Diagnostics Co-operative, Translational and Clinical Research Institute,<br>Clarke, Samantha; North Bristol NHS Trust<br>Deeks, Jonathan; University of Birmingham, Test Evaluation Research Group, Institute of Applied Health Research,<br>Drake, Marcus; North Bristol NHS Trust<br>Drinnan, Michael; Newcastle Upon Tyne Hospitals NHS Trust, Medical Physics; Newcastle Upon Tyne Hospitals NHS Foundation Trust, Translational and Clinical Research Institute<br>Dyer, Calie; Cardiff University, Centre for Trials Research<br>Hood, Kerenza; Cardiff University,<br>Joseph-Williams, Natalie; Cardiff University, Cochrane Institute of Primary Care and Public Health<br>Marsh, Lucy; Cardiff University, Centre for Trials Research<br>Milosevic, Sarah; Cardiff University, Centre for Trials Research<br>Pickard, Robert; Newcastle Upon Tyne Hospitals NHS Trust, Department of Urology<br>Schatzberger, Tom; NHS Northumberland Clinical Commissioning Group, Corbridge Health Centre<br>Takwoingi, Yemisi; University of Birmingham, Test Evaluation Research Group, Institute of Applied Health Research,<br>Harding, Chris; Newcastle Upon Tyne Hospitals NHS Foundation Trust, Department of Urology<br>Edwards, Adrian; Cardiff University, Division of Population Medicine
Keywords:	PRIMARY CARE, UROLOGY, Adult urology < UROLOGY

SCHOLARONE™
Manuscripts

I, the Submitting Author has the right to grant and does grant on behalf of all authors of the Work (as defined in the below author licence), an exclusive licence and/or a non-exclusive licence for contributions from authors who are: i) UK Crown employees; ii) where BMJ has agreed a CC-BY licence shall apply, and/or iii) in accordance with the terms applicable for US Federal Government officers or employees acting as part of their official duties; on a worldwide, perpetual, irrevocable, royalty-free basis to BMJ Publishing Group Ltd ("BMJ") its licensees and where the relevant Journal is co-owned by BMJ to the co-owners of the Journal, to publish the Work in this journal and any other BMJ products and to exploit all rights, as set out in our licence.

The Submitting Author accepts and understands that any supply made under these terms is made by BMJ to the Submitting Author unless you are acting as an employee on behalf of your employer or a postgraduate student of an affiliated institution which is paying any applicable article publishing charge ("APC") for Open Access articles. Where the Submitting Author wishes to make the Work available on an Open Access basis (and intends to pay the relevant APC), the terms of reuse of such Open Access shall be governed by a Creative Commons licence – details of these licences and which Creative Commons licence will apply to this Work are set out in our licence referred to above.

Other than as permitted in any relevant BMJ Author's Self Archiving Policies, I confirm this Work has not been accepted for publication elsewhere, is not being considered for publication elsewhere and does not duplicate material already published. I confirm all authors consent to publication of this Work and authorise the granting of this licence.

For peer review only

Protocol for PRImary care Management of lower Urinary tract Symptoms in men: Protocol for development and validation of a diagnostic and clinical decision support tool (The PriMUS Study).

Title: Protocol for PRImary care Management of lower Urinary tract Symptoms in men: Protocol for development and validation of a diagnostic and clinical decision support tool (The PriMUS Study).

Bethan Pell[1] **(corresponding author)**, Emma Thomas-Jones[1], Alison Bray[2,3], Ridhi Agarwal[8], Haroon Ahmed[5], Joy Allen[4], Samantha Clarke[7], Jon Deeks[8], Marcus Drake[7], Michael Drinnan[2,3], Calie Dyer[1], Kerenza Hood[1], Natalie Joseph-Williams[5], Lucy Marsh[1], Sarah Milosevic[1], Rob Pickard[4], Tom Schatzberger[9], Yemisi Takwoingi[8], Chris Harding[4], Adrian Edwards[5]

Author Affiliations

[1]Centre for Trials Research, Cardiff University, Heath Park, Cardiff CF14 4YS, UK

[2]Medical Physics, Newcastle Upon Tyne NHS Hospital Trust, Newcastle Freeman Hospital, Freeman Rd, High Heaton, Newcastle upon Tyne NE7 7DN

[3]Translational and Clinical Research Institute, Newcastle University, Newcastle upon Tyne, NE1 7RU

[4]Department of Urology, Newcastle Upon Tyne NHS Hospital Trust, Newcastle Freeman Hospital, Freeman Rd, High Heaton, Newcastle upon Tyne NE7 7DN

[5]Division of Population Medicine, School of Medicine, Neuadd Meirionnydd, Cardiff University, Cardiff CF14 4YS, UK

[6]National Institute for Health Research (NIHR) Newcastle In Vitro Diagnostics Co-operative, Translational and Clinical Research Institute, Medical School, Newcastle University, Newcastle upon Tyne NE2 4HH

[7] North Bristol NHS Trust, Southmead Hospital, Southmead Road, Westbury-on-Trym, Bristol, BS10 5NB

For peer review only

Protocol for PRImary care Management of lower Urinary tract Symptoms in men: Protocol for development and validation of a diagnostic and clinical decision support tool (The PriMUS Study).

[8] Test Evaluation Research Group, Institute of Applied Health Research, University of Birmingham, Birmingham, B15 2TT

[9] Corbridge Health Centre, NHS Northumberland Care Commissioning Group, Newcastle Road, Corbridge, NE45 5LG

Corresponding Author Information

Bethan Pell pellb@cardiff.ac.uk

Address: 7th floor Neuadd Meirionnydd, Heath Park, Cardiff CF14 4YS

Word Count

4280

For peer review only

Protocol for PRImary care Management of lower Urinary tract Symptoms in men: Protocol for development and validation of a diagnostic and clinical decision support tool (The PriMUS Study).

ABSTRACT

Introduction: Lower Urinary Tract Symptoms (LUTS) is a bothersome condition affecting older men that can lead to poor quality of life. General Practitioners (GPs) currently have no easily available assessment tools to help effectively diagnose causes of LUTS and aid discussion of treatment with patients. Men are frequently referred to urology specialists who often recommend treatments that could have been initiated in Primary Care. GP access to simple, accurate tests and clinician decision tools are needed to facilitate accurate and effective patient management of LUTS in primary care.

Methods and analysis: PriMUS is a prospective diagnostic accuracy study based in primary care. The study will determine which of a number of index tests used in combination, best predict three urodynamic observations in men who present to their GP with LUTS. These are detrusor overactivity, bladder outlet obstruction, and/or detrusor underactivity. Two cohorts of participants, one for development of the prototype diagnostic tool, and one for validation, will undergo a series of simple index tests and the invasive reference standard (invasive urodynamics). We will develop and validate three diagnostic prediction models based on each condition, and then combine them with management recommendations to form a clinical decision support tool.

Ethics and dissemination: Ethics approval is from the Wales Research Ethics Committee 6. Findings will be disseminated through peer-reviewed journals and conferences; results will be of interest to professional and patient stakeholders.

Study registration: ISRCTN10327305

Strengths and limitations of this study

- Prospective, multicentre study in an appropriate population in primary care.
- The index tests are tests that can be done routinely in primary care or at home by patients.
- The diagnostic models developed will be validated in a separate cohort of men from the same population.
- The assumed prevalence of the three target conditions may be different in practice.
- Some test results may be missing or difficult to obtain.

Protocol version: 7.0

Keywords: primary care, urology, adult urology

For peer review only

Protocol for PRImary care Management of lower Urinary tract Symptoms in men: Protocol for development and validation of a diagnostic and clinical decision support tool (The PriMUS Study).

INTRODUCTION

Lower urinary tract symptoms (LUTS), such as frequent urination, a slow stream, and having to wake in the night to urinate, affect a significant proportion of older men and can lead to poor quality of life. Three common causes of LUTS are: instability of the bladder muscle (detrusor overactivity, DO), benign enlargement of the prostate gland causing bladder outlet obstruction (BOO), and weakness of the bladder muscle (detrusor underactivity, DU). These may be present individually or in combination.

The reference standard test for investigation of LUTS, and thus diagnosis of DO, BOO and DU, is invasive urodynamics, which takes place in secondary care. It involves insertion of catheters into the patient's bladder and rectum so that the behaviour of the bladder and outlet can be examined during filling and voiding. Owing to availability, complexity and cost, management decisions for men with LUTS are usually based on results from a combination of non- and minimally-invasive investigations instead. These include digital rectal examination (DRE) to assess prostate size, symptoms questionnaires, uroflowmetry, and measurement of post void residual.

NICE Guidelines suggest that many men referred to specialist care with LUTS are eventually managed conservatively, and so could have remained within primary care. Male LUTS account for around four presentations per month in an average-sized General Practitioner (GP) practice. This rate of presentation, although high enough to represent a large burden

For peer review only - http://bmjopen.bmj.com/site/about/guidelines.xhtml

on the National Health Service (NHS), makes it difficult for GPs to gain sufficient expertise to be confident about diagnosis and management. Further, GPs don't have access to simple tools giving an indication of the most likely cause of symptoms to guide treatment and management. Making such a tool available should improve treatment efficacy, standardise treatment, reduce unnecessary referrals, expedite referral of those requiring specialist care, and thus improve cost-effectiveness of NHS care.

This led to the National Institute for Health Research (NIHR) releasing a 2015 health technology assessment (HTA) commissioned call (HTA number 15/40) seeking the delivery of: *The development of a decision aid to help inform the choice of treatment or need for specialist referral for men presenting with lower urinary tract symptoms in primary care*. Our team was successful in obtaining this funding and here we describe the protocol for our study: *Primary care management of lower urinary tract symptoms in men: Development and validation of a diagnostic and clinical decision support tool (The PriMUS Study)*.

Aims, Objectives and Outcome measures

The PriMUS study aims to develop three diagnostic prediction models based on the results of simple clinical tests that can provide a useful prediction of urodynamic observations. We will assess the diagnostic accuracy of these models, which will be implemented in software, along with management recommendation algorithms to forma clinical decision support tool for use in UK primary care. Our primary and secondary objectives and measures are outlined below.

Primary Objectives

- Develop a statistical model to predict the likelihood of three urological conditions (bladder outlet obstruction, detrusor overactivity, detrusor underactivity) based on a series of non-invasive index tests, with invasive urodynamics as the reference standard
- Estimate the diagnostic accuracy of the above statistical model in an independent validation cohort.

Secondary Objectives

- Develop a series of patient management recommendations and thresholds for clinically useful diagnostic prediction by expert consensus and with reference to current clinical guidelines that map to the diagnoses predicted by the statistical model.
- Combine the statistical model and management recommendations into an online tool that will form the prototype clinical decision support tool.

For peer review only - http://bmjopen.bmj.com/site/about/guidelines.xhtml

Protocol for PRImary care Management of lower Urinary tract Symptoms in men: Protocol for development and validation of a diagnostic and clinical decision support tool (The PriMUS Study).

- Complete a qualitative study to explore the feasibility of introducing the clinical decision support tool into primary care including potential acceptability to primary care staff and patients.
- Collect NHS costs involved in delivering the new pathway and compare with cost of standard pathway calculated from NHS and other sources.

Primary outcome

Sensitivity and specificity of the diagnostic models for detecting detrusor underactivity, bladder outlet obstruction and detrusor over activity will be determined. The three conditions will be coded as binary outcomes (present/absent).

Secondary outcomes

- A patient management algorithm to guide initial treatment for men with LUTS
- A prototype online clinical decision support tool for use in primary care
- Qualitative summary of patients' and clinicians' views on the use of a LUTS clinical decision support tool in the primary care setting
- Costs / savings of implementation of the primary care LUTS clinical decision support tool both from a population and individual patient perspective

METHODS AND ANALYSIS

Study design

This is a prospective, diagnostic accuracy study involving the development and validation of a diagnostic tool. An internal pilot will assess primary care recruitment, acceptability of the reference test (invasive urodynamics) and data collection. Two cohorts of participants, one for development of the prototype diagnostic tool, and one for validation, will undergo a series of index tests and the invasive reference standard (urodynamics) in approximately 90 GP practices across Newcastle Upon Tyne, Wales and Bristol (a list of study sites can be found on ISRCTN). There will also be qualitative data collection to explore acceptability of the urodynamics test, develop management recommendations for the tool and for user-testing of the prototype.

Participants

Adult men who consult their GP with one or more LUTS in UK primary care settings.

Inclusion criteria

- Men aged 16 years and over.
- Men who present to their GP with a complaint of one or more bothersome lower urinary tract symptoms (this includes men on current treatment, but who are still symptomatic).
- Men able and willing to give informed consent for participation in study.

For peer review only

Protocol for PRImary care Management of lower Urinary tract Symptoms in men: Protocol for development and validation of a diagnostic and clinical decision support tool (The PriMUS Study).

- Men able and willing to undergo all index tests and reference test, and complete study documentation.

Exclusion criteria

- Men with neurological disease or injury affecting lower urinary tract function.
- Men with LUTS considered secondary to current or past invasive treatment or radiotherapy for pelvic disease.
- Men with contraindications to urodynamics such as heart valve or joint replacement surgery within the last three months, or immunocompromised/immunosuppressed men.
- Men with indwelling urinary catheters or who carry out intermittent self-catheterisation.
- Men whose initial assessment suggests that clinical findings are suggestive of:
  - Prostate or bladder cancer. If later deemed unlikely, they become eligible for study participation.
  - Recurrent or persistent symptomatic urinary tract infection (UTI). If this is successfully treated but LUTS remain, they become eligible for study participation.
  - Urinary retention, for example palpable bladder after voiding.
- Men unable to consent in English or Welsh where a suitable translator is not available.

For peer review only

Test Selection

Test selection, including the reference standard, was informed by a systematic review included in the relevant NICE guideline CG97[1] updated with a study-specific unpublished selective review by our group in 2015, the judgement of the expert clinical members of the study team, and the stipulations of the funding commissioning brief. All participants undergo all tests, which are a combination of:

- Tests carried out for eligibility assessment prior to enrolment, as described above.
- Tests carried out at participant visits to a primary or secondary care location for the purpose of the study following enrolment.
- Tests carried out by the participant at home following enrolment.

Index Tests

Protocol for PRImary care Management of lower Urinary tract Symptoms in men: Protocol for development and validation of a diagnostic and clinical decision support tool (The PriMUS Study).

Twelve potential parameters will be considered for the three logistic regression models. The investigations that provide these parameters are described in Table 1 below.

Table 1. Index Tests and Input parameters that will be tested for use in the three logistic regression models.

Test	*Result*	*Input parameters that will be tested for use in the three logistic regression models. (result or unit)*
Relevant demographics	Age in years	Age (years)
Physical examination of abdomen	Bladder palpable/not palpable	N/A
Digital rectal examination	Prostate mild/moderate/severe enlargement<br>Further assessment for prostate cancer required/not required	Prostate size (enlarged/not enlarged)
Prostate specific antigen	PSA value – established thresholds for further assessment for prostate cancer (typically > 3 ng/ml) or benign enlargement (typically ≥ 1.5 ng/ml)<br>For clinical decision support tool: continuous variable in ng/mL	Prostate specific antigen (ng/ml)
International Consultation on Incontinence Questionnaire Male Lower Urinary Tract Symptoms Short Form (ICIQ M-LUTS)	Total score (0-52); Voiding symptom score (0-20), storage symptom score (0-24). Individual symptom bother scores scored separately from symptom severity scores (0-130).	Storage/incontinence symptoms sub-score<br>Voiding symptoms sub-score
International Prostate Symptom Score (IPSS) questionnaires.	Total score (0-35)	Storage/incontinence symptoms sub-score<br>Voiding symptoms sub-score
Bladder Diary	Waking (day) time frequency, sleeping (night) time frequency, 24 hour voided volume, daytime voided volume, nocturnal voided volume, average volume voided each void, total urgency scores	Mean urgency score<br>Mean 24-hour fluid intake (ml)
Uroflowmetry (Flowtaker)	Maximum flow rate, voided volume against normal age-adjusted range. Single value in ml/s	Median maximum flow rate (ml/s)<br>Median voided volume (ml)<br>Mean 24-hour frequency<br>Mean nocturia
Post void residual	Residual volume against normal age-adjusted range. Single value in ml	Post void residual volume (ml)

Reference Standard

For peer review only - http://bmjopen.bmj.com/site/about/guidelines.xhtml

Protocol for PRImary care Management of lower Urinary tract Symptoms in men: Protocol for development and validation of a diagnostic and clinical decision support tool (The PriMUS Study).

Our reference standard is invasive urodynamics, a test routinely carried out in a specialist care setting for the investigation of LUTS. For this study, it will be performed using portable equipment (Goby, Laborie, Mississauga, Canada) in either a primary or secondary care location, by specially-trained urodynamic nurses, according to International Continence Society (ICS) standards[2]. Safety information is covered in the *Safety* section below.

Study Procedures

Data Collection

GPs, primary care nurses or an appropriately trained delegate will undertake the data collection relating to all the index tests. Specialist trained urodynamic nurses will undertake the data collection for the reference test.

Data Management

All data collection will be by electronic data capture using a bespoke database developed by the Cardiff University Centre for Trials Research Clinical Trials Unit (CTR), and paper copies of all case report forms (CRFs) will be available.

Identification and Screening

All men will be identified either opportunistically during a GP consultation, or by regular, pre-defined primary care database searches. They must undergo three screening tests prior to enrolment into the study; a physical examination of the abdomen (palpable bladder check), DRE and Prostate Specific Antigen (PSA) test. The latter two test results are accepted if they have undergone these investigations within the last six months.

Informed Consent

Informed consent will be obtained in the first study visit (Study Visit Part A) prior to any study procedures by those suitably trained and on the delegation log. Eligible patients will be given time to consider before being asked to sign the consent form. Once consented, participants will be allocated a unique study number (participant ID).

Separate informed consent will be taken for participation in the qualitative data collection.

Withdrawal

Patients will be notified that they can withdraw consent for their participation in the study at any time during the study period.

Study Visit Part A

Once informed consent is obtained, the remaining index tests will be collected. This includes a baseline assessment (collecting demographic information, relevant medication and medical history) and two self-reported questionnaires; IPSS and ICIQ. Participants will be

For peer review only

Protocol for PRImary care Management of lower Urinary tract Symptoms in men: Protocol for development and validation of a diagnostic and clinical decision support tool (The PriMUS Study).

given the bladder diary to complete for three days at home and instructed to bring this to their invasive urodynamic visit (Study Visit Part B).

Study Visit Part B - Reference standard

On arrival, the patient will be asked to pass urine into a flowmeter in private, after which a measurement of post void residual ultrasound (one of the index tests; see below) will be made. A dual lumen catheter (one channel to fill the bladder, and the other to measure intravesical pressure, P_{ves}) will be inserted into the bladder via the urethra, and a single lumen catheter inserted into the rectum to measure abdominal pressure (P_{abd}). Detrusor pressure (P_{det}), generated by the bladder muscle itself, is calculated by subtracting P_{abd} from P_{ves}.

Filling Phase

The patient will be asked to bring their completed bladder diary (one of the index tests; see above) to their urodynamics appointment, providing the urodynamic nurse with an indication of their maximum bladder capacity. The patient's bladder will be filled with sterile saline at a maximum rate of 50 ml/min. They will be asked to report the first sensation of bladder filling, followed by the point at which they feel the normal desire to void, and finally the strong desire to void. At this point bladder filling will be stopped and provocation, in the form of running taps and asking the patient to cough, will be performed.

Voiding Phase

Following provocation, the patient will be given permission to void, marking the start of the voiding phase. Voided volume (V_{void}) and flow rate (Q) will be measured as they pass urine into the flowmeter.

If either the filled or voided volumes are below 150 ml, the filling and voiding phases will be repeated once more using a maximum filling rate of 20 ml/min.

Diagnostic Definitions

For peer review only - http://bmjopen.bmj.com/site/about/guidelines.xhtml

Protocol for PRImary care Management of lower Urinary tract Symptoms in men: Protocol for development and validation of a diagnostic and clinical decision support tool (The PriMUS Study).

Definition of our three target conditions will be based upon the following parameters measured during invasive urodynamics and subsequently read from a graphical representation of the test:

1. Maximum detrusor contraction pressure during the filling phase.
2. Maximum flow rate during the voiding phase (Q_{max}).
3. Detrusor pressure at the point of maximum flow rate ($P_{detQmax}$).

If there are no detrusor contractions during filling, DO is not present. If there are any contractions (contraction pressure>0), DO is present.

Diagnosis of BOO is based upon the bladder outlet obstruction index (BOOI), defined as PdetQmax-2*Qmax. BOO is present if BOOI>40, and absent if BOOI≤40.

Diagnosis of DU is based upon the bladder contractility index (BCI), defined as PdetQmax+5*Qmax. DU is present if BCI<100, and absent if BCI≥100.

Debrief process and monitoring process

The urodynamic nurses will debrief the patient following the urodynamic procedure providing them with a post urodynamics leaflet and safety card. The urodynamic nurse will also instruct the patient that they will receive a 3 Day Follow Up Phone Call, to monitor for any related adverse events.

3 Day Follow Up Phone Call

The urodynamic nurses will contact the patient 3 days (+/- flexibility if phone call falls on weekend) after their urodynamic procedure to monitor for any adverse events and Serious Adverse Events (SAEs). Any SAEs are subsequently recorded by the urodynamic nurses and processed centrally by CTR. This process is outlined in the Safety Section (see below).

Review Process

Invasive urodynamics is a complex investigation and interpretation can be challenging. Further, because standard practice involves interaction between the reference and some index tests as described earlier, their primary interpretation in this study is not blinded.

For peer review only

Protocol for PRImary care Management of lower Urinary tract Symptoms in men: Protocol for development and validation of a diagnostic and clinical decision support tool (The PriMUS Study).

Therefore, a review process will be implemented to ensure the integrity of the reference standard. All studies will be second-read by a blinded reviewer to extract the three parameters above. If any of the resulting diagnoses differ between the nurse and this reviewer, the case will go to a second non-blinded reviewer who makes the final decision.

Uroflowmetry* (Flowtaker)

The patient is provided with the Flowtaker at the end of their invasive urodynamic visit. They will be provided with an information sheet on how to use this and instructed to not start this until given the greenlight to do so during their 3 Day Follow Up Phone Call. The Flowmeter will be given with a pre-paid envelope for the patient to post back to their urodynamic nurse for data upload.

GP Summary Report

Once the reference and index tests have taken place, results are compiled into a report which is provided to GPs, along with a summary of relevant NICE-recommended managements[1], to help inform management of the patient.

6 Month Follow Up

A review of the patient's medical notes will take place 6 months after the patient's treatment and management decision with the GP. This will include any changes to treatment, or management and whether they have since been referred to secondary care. Figure 1 depicts a flow diagram of the patient pathway.

[INSERT FIGURE 1 HERE]

Safety and Pharmacovigilance

Invasive urodynamics has the potential to cause adverse events. A medical doctor will be required on site whilst the test is taking place. Due to a 5% risk of a urinary tract infection[3], the urodynamic nurse will also provide the patient with a post-urodynamics debrief sheet following the test, informing them on the importance of drinking plenty of water for 24 hours following the test, how to identify signs of an infection, and to seek medical care if they suspect they have one.

For peer review only - http://bmjopen.bmj.com/site/about/guidelines.xhtml

Adverse events will be captured by the urodynamic nurses either during Study Visit Part B, or during the 3 day follow up phone call. For serious adverse events (SAEs), an assessment of causality between the event and the study intervention, and the expectedness of the event, will be carried out by the carried out by the PI, or delegated urodynamic nurse, and then independently by a clinical reviewer. If the clinical reviewer classes the event as *probably* or *definitely* caused by the intervention, it will be classified as a serious adverse reaction.

Sample Size

Sample size calculations were carried out separately for the model development and validation cohorts. For both we used estimated prevalences for DO, BOO and DU of 57%, 31% and 16% respectively based on previous literature[4,5] and clinical expertise.

Development Cohort

The sample size for developing our predictive models was based on a rule of thumb suggesting that five events per variable are required[6]. We chose a sample size of 350 to allow at least 11 variables in each model. This was driven by our lowest estimated prevalence of 16% for DU, giving 56 events (DU diagnoses).

Validation Cohort

The sample size for the validation cohort was chosen to ensure that estimates of test accuracy are made with adequate precision. We deem sensitivity and specificity of 75% to be the minimum clinically useful performance. We chose a sample size of 325, giving estimates of sensitivity of 75% to within 8%, 10% and 14% for DO, BOO and DU respectively, based on ‘positive’ samples of 185, 101 and 52, and estimates of specificity of 75% to within 8%, 7% and 6%, based on ‘negative’ samples of 140, 224 and 273. Better sensitivity and specificity will give narrower confidence intervals.

Attrition

In order to allow an attrition rate of 20-25%, the resulting total of 675 was increased to give a final sample size of 880.

STATISTICAL ANALYSIS

For peer review only

Protocol for PRImary care Management of lower Urinary tract Symptoms in men: Protocol for development and validation of a diagnostic and clinical decision support tool (The PriMUS Study).

Model development will be performed using results from the first 350 datasets, and external model validation performed using the subsequent 325 datasets.

Model Development

Candidate predictor variables will be selected from those listed in Table 2. Their selection has been informed by subject knowledge using literature review and expert judgement. As predictor distributions should be wide to facilitate reliable predictions, we will explore the distribution of each predictor prior to selection. Relationships between predictors will also be investigated; where indicated we will group related variables into a composite variable or exclude if highly correlated with other variables. Candidate predictors will not be selected based on univariable analyses; this practice is discouraged because predictors that may be important in a multivariable model can be missed and may also lead to overoptimistic models. Therefore, all selected candidate predictor variables will be included in the multivariable logistic regression models without evaluations of association between outcome and predictor and assessment of statistical significance. To gain maximum diagnostic information, continuous variables will not be categorised. We will allow for non-linearity by using a multivariable fractional polynomial (MFP) approach to identify appropriate transformations. This may lead to the inclusion of non-linear terms in the models thus increasing the number of variables in the models. Using multiply imputed data and Rubin's rule, we will develop each model using backward elimination with a p value of 0.157 to select predictors for inclusion in each model. We chose this p value because it is known to be a good proxy for the Akaike information criterion (AIC) approach. If the repeated use of Rubin's rule is computationally challenging, we will use the approximation to Rubin's rule recommended by Wood et al (2008)[7].

Model Validation

The predictive performance of each model will be assessed in terms of discrimination, that is the ability to distinguish between those who do or do not have a particular diagnosis, and calibration meaning agreement between predicted and observed probabilities. Discriminative ability will be assessed using the c-index and its 95% confidence intervals. For a logistic model, this is equivalent to the area under the ROC curve (AUC). Calibration will be evaluated in two ways. Calibration plots of average observed probability against predicted probability will be used to visually assess calibration. Within each quintile or decile of predicted probability (depending on the distribution of data), the average predicted probability will be compared with the corresponding observed proportions. We will also quantify calibration by estimating the calibration slope of the prognostic index (linear predictor) using logistic regression with the linear predictor as the covariate.

The apparent c-index and calibration slope will be estimated for each model. Bootstrapping will be used for internal validation to assess model overfitting and optimism. For each model,

For peer review only - http://bmjopen.bmj.com/site/about/guidelines.xhtml

we will obtain 100 bootstrap samples from each imputed dataset and repeat the variable selection process. The optimism is the difference between the c-index from the bootstrap sample and that from the original imputed dataset. The average optimism will be determined across bootstrap samples and imputed datasets, and the optimism-adjusted c-index will be calculated by subtracting the average optimism from the apparent c-index of the original model. Similarly, we will obtain the optimism adjusted calibration slope. The optimism-adjusted calibration slope will be used as the uniform shrinkage factor to correct a model.

We will externally validate the models and calculate performance statistics (c-index and calibration slope) using the validation cohort. The value for the calibration slope should ideally be one signifying perfect agreement between the predicted probabilities and the observed probabilities. A calibration slope < 1 indicates that a model over-predicts while a calibration slope > 1 indicates under prediction. From the qualitative research we will ascertain distributions of probability (risk) thresholds for clinical usefulness of the prediction in guiding treatment of each condition. The sensitivity and specificity (and their corresponding 95% confidence intervals), will be calculated for these risk thresholds and plotted on an ROC plot for each model.

Missing Data

Patterns of missing data will be investigated to infer the ease with which each parameter can be obtained in practice. In the event of missing data, multiple imputation by chained equations[8] will be used to impute missing values in order to avoid bias and make best use of the data.

SECONDARY SUB-STUDIES

Table 2. Secondary Sub-Studies

Qualitative Studies	Details
Patient and Clinician Acceptability Interviews	During the pilot phase we will conduct qualitative interviews with both patients and clinicians to assess the acceptability of the urodynamic procedure and PriMUS Study, as part of our progression criteria.

For peer review only

Protocol for PRImary care Management of lower Urinary tract Symptoms in men: Protocol for development and validation of a diagnostic and clinical decision support tool (The PriMUS Study).

Development of Management Recommendations	Algorithms are required to link outputs from the statistical models, which will be likelihoods of each target condition, with patient management recommendations to form the clinical decision support tool. The starting point will be recommendations from the relevant NICE clinical guideline. Qualitative work with urologists will support the development of these management recommendations, through posing clinical case scenarios to urologists using interview and questionnaire methodologies.
Tool Feasibility Assessment	The aim of the user-testing phase will be to build on the interviews conducted as part of the pilot phase evaluation to assess participants' attitudes and reactions to the proposed tool, in terms of content, design, and perceived acceptability and feasibility of using the tool in primary care settings. Feedback will be used to improve and refine the tool. We will conduct user testing with GPs.

STUDY MANAGEMENT

The study is sponsored by Cardiff University, coordinated by CTR and co-led by The Newcastle upon Tyne Hospitals NHS Foundation Trust (NuTH). The other partner organisations will be Birmingham University, University of Bristol, and North of England Commissioning Support.

Study management Group

The Study Management Group (SMG) will meet monthly throughout the course of the study and will include the Chief Investigators, co-applicants, collaborators, study manager, data manager and administrator. SMG members will be required to sign up to the remit and conditions as set out in the SMG Charter.

Study Steering Committee

For peer review only - http://bmjopen.bmj.com/site/about/guidelines.xhtml

Protocol for PRImary care Management of lower Urinary tract Symptoms in men: Protocol for development and validation of a diagnostic and clinical decision support tool (The PriMUS Study).

An independent Study Steering Committee (SSC) consisting of an independent chairperson, two independent members and a patient representative will provide oversight of The PriMUS Study. Instead of a separate Independent Data Monitoring Committee (IDMC), the SSC will also provide oversight of all matters relating to patient safety and data quality. Members will be required to sign up to the remit and conditions as set out in the SSC Charters and will meet annually.

ETHICS AND DISSEMINATION

Research approvals

The Wales REC 6 has approved the study (17/WA/0155) on the 20th June 2017 and subsequent R&D Approval for Wales on 21st August 2017 and HRA Approval on 23rd August 2017.

All study participants will give informed consent before taking part (see earlier).

The following substantial amendments were made to the trial and were communicated to all trial sites: Substantial Amendment 1 (3rd October 2017); Substantial Amendment 2 (10th January 2018); Substantial Amendment 3 (20th April 2018); Substantial Amendment 4 (26th February 2019); Substantial Amendment 5 (6th June 2019); Substantial Amendment 6 (6th September 2019)

The study has the following registration: **ISRCTN10327305**

Dissemination plan

Following completion of the study, a final report will be prepared for the National Institute of Health Research (NIHR) Journal series. A paper describing the primary results will be submitted to a high impact, international, peer-reviewed journal. Qualitative studies and sub-studies will also be submitted for publication. We will present our findings at national and international scientific meetings.

With the assistance of our collaborators and lay representatives we will disseminate the study findings to a wide NHS and general audience and promote uptake of outputs into clinical care. This will include presentations at meetings and written executive summaries for key stakeholder groups such as Primary Care Trusts, Secondary Care Trusts, Health Boards, Royal Colleges, Medical Schools, and relevant patient groups.

All publications and presentations relating to the study will be authorised by the SMG in accordance with the study's publication policy.

Competing Interests Statement

For peer review only

Protocol for PRImary care Management of lower Urinary tract Symptoms in men: Protocol for development and validation of a diagnostic and clinical decision support tool (The PriMUS Study).

One of the index tests, Flowtaker, was developed by a team from NuTH and Newcastle University, including two individuals who are grant co-applicants, members of the study management team and co-authors (Authors 3 and 10). In 2014 the device was licensed to MMS (Enschede, The Netherlands) and royalties from the sale of the device were paid to NuTH (not to the individuals). MMS was subsequently acquired by Laborie who removed Flowtaker from the market in January 2018.

Author Contributions

AE and CH are Co-Chief Investigators of this study. AE and CH led the development of the research question, study design, obtaining the funding, and implementation of the study protocol, along with JA, AB, MD, JD, MD, KH, YT and ETJ. BP is the Study Manager and ETJ the Senior Study Manager who coordinated the operational delivery of the study protocol and recruitment. SC provides research nurse insight and support. YT and RA are the study statisticians. CD and LM are the data managers. All authors listed provided critical review and final approval of the manuscript.

Funding

This study is funded by the NIHR (Health Technology Assessment programme), funder reference 15-40-05.

Disclaimer

The views expressed are those of the authors and not necessarily those of the NHS, the NIHR or the Department of Health and Social Care. Neither the Sponsor nor the Funder had any role on the study design; collection, management, analysis and interpretation of data; writing of this manuscript or in the decision to submit this manuscript for publication.

Sponsor

Cardiff University, Research and Innovation Services Department, Contracts Team, Cardiff University, 30-36 Newport Road, Cardiff, CF24 0DE. Contact person: Ms Helen Falconer; FalconerHE@cardiff.ac.uk. Sponsor reference: SPON 1553-16

Acknowledgements

For peer review only

Protocol for PRImary care Management of lower Urinary tract Symptoms in men: Protocol for development and validation of a diagnostic and clinical decision support tool (The PriMUS Study).

Professor Robert Pickard † for development of the research question, study design, obtaining the funding, and implementation of the study protocol. The authors would like to thank the research nurses for their support during the study Debra Barnett, Laura Bevan, Jane Davies, Alison Edwards, Gareth Kennard-Holden, Lisa Mellish, Joanne Sullivan, Joanne Thompson.

The Centre for Trials Research receives funding from Health and Care Research Wales and Cancer Research UK.

AJA is supported by the National Institute for Health Research (NIHR) Newcastle In Vitro Diagnostics Co-operative. The views expressed are those of the authors and not necessarily those of the NIHR or the Department of Health and Social Care.

The authors would also like to acknowledge the contribution of the Trial Steering Committee members, namely Professor Tom Fahey, Professor Rafael Perea, Dr Gail Hayward, Dr Ian Pearce and Mr Alan Pryce.

Patient and Public Involvement

The authors would like to acknowledge the contribution of Malcom Gristwood and Ray White as the public and patient involvement representatives on the Study Management Group.

Consent for publication

Not Applicable

Availability of data and material

Not Applicable

REFERENCES

1. National Institute for Health and Clinical Excellence (**NICE**). *The management of lower urinary tract symptoms in men: Clinical guideline 97.* May **2010**.

2. **Drake,** M, Doumouchtsis SK, Hashim H, Gammie A. Fundamentals of urodynamic practice, based on International Continence Society good urodynamic practices recommendations. 2018. Neurourol Urodyn. 2018 Aug;37(S6):S50-S60. doi: 10.1002/nau.23773.

3. **Foon** R, Toozs-Hobson P, Latthe P. Prophylactic antibiotics to reduce the risk of urinary tract infections after urodynamic studies. Cochrane Database of Systematic Reviews **2012**, Issue 10. Art. No.: CD008224. DOI: 10.1002/14651858.CD008224.pub2.

† Deceased 24 July 2018

For peer review only - http://bmjopen.bmj.com/site/about/guidelines.xhtml

Protocol for PRImary care Management of lower Urinary tract Symptoms in men: Protocol for development and validation of a diagnostic and clinical decision support tool (The PriMUS Study).

4. Kuo H. C. Videourodynamic analysis of pathophysiology of men with both storage and voiding lower urinary tract symptoms. Urology 2007; 70: 272–276.

5. **van Doorn** B, Blanker MH, Kok ET, Westers P, Bosch JLHR. Prevalence, incidence, and resolution of nocturnal polyuria in a longitudinal community-based study in older men: The Krimpen study. European Urology 2013; 63: 542-554

6. **Vittinghoff** E, McCulloch CE. *Relaxing the rule of ten events per variable in logistic and Cox regression. Am J Epidemiol.* **2007**;165(6):710-8.

7. **Wood** AM, White IR, Royston P. How should variable selection be performed with multiply imputed data? Statistics in Medicine. 2008;27:3227–3246.

8. **van Buuren** S, Boshuizen HC, Knook DL. *Multiple imputation of missing blood pressure covariates in survival analysis.* Stat Med **1999**;18(6):681-94.

For peer review only

Figure 1 – Patient Pathway

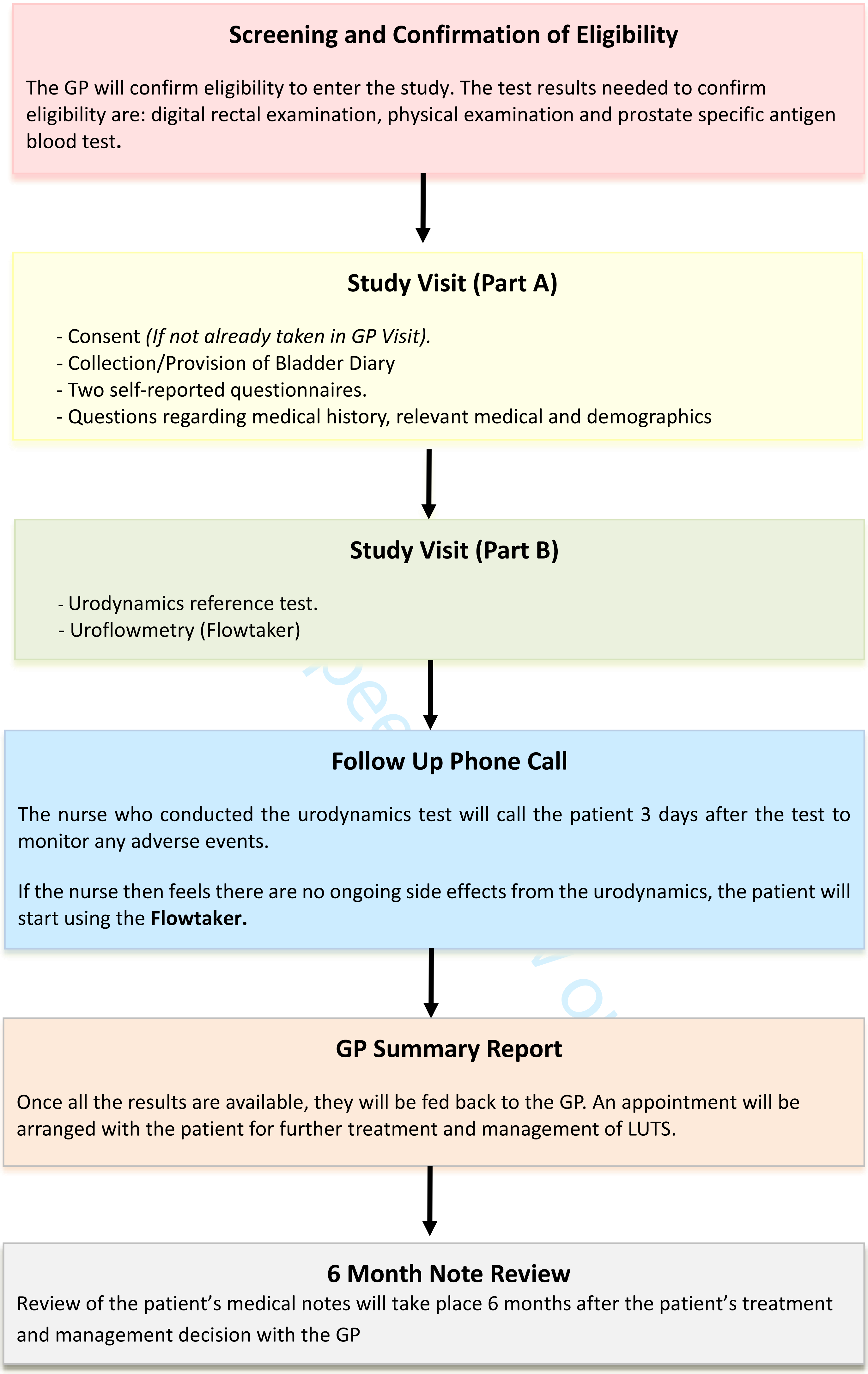

TRIPOD Checklist: Prediction Model Development and Validation

Section/Topic	Item		Checklist Item	Page
**Title and abstract**				
Title	1	D;V	Identify the study as developing and/or validating a multivariable prediction model, the target population, and the outcome to be predicted.	1
Abstract	2	D;V	Provide a summary of objectives, study design, setting, participants, sample size, predictors, outcome, statistical analysis, results, and conclusions.	1
**Introduction**				
Background and objectives	3a	D;V	Explain the medical context (including whether diagnostic or prognostic) and rationale for developing or validating the multivariable prediction model, including references to existing models.	2
	3b	D;V	Specify the objectives, including whether the study describes the development or validation of the model or both.	2-3
**Methods**				
Source of data	4a	D;V	Describe the study design or source of data (e.g., randomized trial, cohort, or registry data), separately for the development and validation data sets, if applicable.	3
	4b	D;V	Specify the key study dates, including start of accrual; end of accrual; and, if applicable, end of follow-up.	N/A
Participants	5a	D;V	Specify key elements of the study setting (e.g., primary care, secondary care, general population) including number and location of centres.	3
	5b	D;V	Describe eligibility criteria for participants.	3-4
	5c	D;V	Give details of treatments received, if relevant.	4-5
Outcome	6a	D;V	Clearly define the outcome that is predicted by the prediction model, including how and when assessed.	3
	6b	D;V	Report any actions to blind assessment of the outcome to be predicted.	
Predictors	7a	D;V	Clearly define all predictors used in developing or validating the multivariable prediction model, including how and when they were measured.	4
	7b	D;V	Report any actions to blind assessment of predictors for the outcome and other predictors.	4
Sample size	8	D;V	Explain how the study size was arrived at.	8
Missing data	9	D;V	Describe how missing data were handled (e.g., complete-case analysis, single imputation, multiple imputation) with details of any imputation method.	9
Statistical analysis methods	10a	D	Describe how predictors were handled in the analyses.	8-9
	10b	D	Specify type of model, all model-building procedures (including any predictor selection), and method for internal validation.	8-9
	10c	V	For validation, describe how the predictions were calculated.	8-9
	10d	D;V	Specify all measures used to assess model performance and, if relevant, to compare multiple models.	8-9
	10e	V	Describe any model updating (e.g., recalibration) arising from the validation, if done.	N/A
Risk groups	11	D;V	Provide details on how risk groups were created, if done.	N/A
Development vs. validation	12	V	For validation, identify any differences from the development data in setting, eligibility criteria, outcome, and predictors.	8-9
**Results**				
Participants	13a	D;V	Describe the flow of participants through the study, including the number of participants with and without the outcome and, if applicable, a summary of the follow-up time. A diagram may be helpful.	6-7
	13b	D;V	Describe the characteristics of the participants (basic demographics, clinical features, available predictors), including the number of participants with missing data for predictors and outcome.	6
	13c	V	For validation, show a comparison with the development data of the distribution of important variables (demographics, predictors and outcome).	N/A
Model development	14a	D	Specify the number of participants and outcome events in each analysis.	8
	14b	D	If done, report the unadjusted association between each candidate predictor and outcome.	N/A
Model specification	15a	D	Present the full prediction model to allow predictions for individuals (i.e., all regression coefficients, and model intercept or baseline survival at a given time point).	N/A
	15b	D	Explain how to the use the prediction model.	N/A
Model performance	16	D;V	Report performance measures (with CIs) for the prediction model.	N/A
Model-updating	17	V	If done, report the results from any model updating (i.e., model specification, model performance).	N/A
**Discussion**				
Limitations	18	D;V	Discuss any limitations of the study (such as nonrepresentative sample, few events per predictor, missing data).	N/A
Interpretation	19a	V	For validation, discuss the results with reference to performance in the development data, and any other validation data.	N/A
	19b	D;V	Give an overall interpretation of the results, considering objectives, limitations, results from similar studies, and other relevant evidence.	N/A
Implications	20	D;V	Discuss the potential clinical use of the model and implications for future research.	N/A
**Other information**				
Supplementary information	21	D;V	Provide information about the availability of supplementary resources, such as study protocol, Web calculator, and data sets.	N/A
Funding	22	D;V	Give the source of funding and the role of the funders for the present study.	11

*Items relevant only to the development of a prediction model are denoted by D, items relating solely to a validation of a prediction model are denoted by V, and items relating to both are denoted D;V. We recommend using the TRIPOD Checklist in conjunction with the TRIPOD Explanation and Elaboration document.

For peer review only

Protocol for PRImary care Management of lower Urinary tract Symptoms in men: Protocol for development and validation of a diagnostic and clinical decision support tool (The PriMUS Study).

Journal:	*BMJ Open*
Manuscript ID	bmjopen-2020-037634.R1
Article Type:	Protocol
Date Submitted by the Author:	18-May-2020
Complete List of Authors:	Pell, Bethan; Cardiff University Centre for Trials Research,<br>Thomas-Jones, Emma; Cardiff University, Centre for Trials Research<br>Bray, Alison; Newcastle Upon Tyne Hospitals NHS Foundation Trust, Medical Physics; Newcastle Upon Tyne Hospitals NHS Foundation Trust, Translational and Clinical Research Institute<br>Agarwal, Ridhi; University of Birmingham, Test Evaluation Research Group, Institute of Applied Health Research,<br>Ahmed, Haroon; Cardiff University, Division of Population Medicine<br>Allen, A; Newcastle University, NIHR In Vitro Diagnostics Co-operative<br>Clarke, Samantha; North Bristol NHS Trust<br>Deeks, Jonathan; University of Birmingham, Public Health, Epidemiology and Biostatistics<br>Drake, Marcus; North Bristol NHS Trust<br>Drinnan, Michael; Newcastle Upon Tyne Hospitals NHS Trust, Medical Physics; Newcastle Upon Tyne Hospitals NHS Foundation Trust, Translational and Clinical Research Institute<br>Dyer, Calie; Cardiff University, Centre for Trials Research<br>Hood, Kerenza; Cardiff University,<br>Joseph-Williams, Natalie; Cardiff University, Cochrane Institute of Primary Care and Public Health<br>Marsh, Lucy; Cardiff University, Centre for Trials Research<br>Milosevic, Sarah; Cardiff University, Centre for Trials Research<br>Pickard, Robert; Newcastle Upon Tyne Hospitals NHS Trust, Department of Urology<br>Schatzberger, Tom; NHS Northumberland Clinical Commissioning Group, Corbridge Health Centre<br>Takwoingi, Yemisi; University of Birmingham, Test Evaluation Research Group, Institute of Applied Health Research,<br>Harding, Chris; Newcastle Upon Tyne Hospitals NHS Foundation Trust, Department of Urology<br>Edwards, Adrian; Cardiff University, Division of Population Medicine
**Primary Subject Heading**:	General practice / Family practice
Secondary Subject Heading:	Urology
Keywords:	PRIMARY CARE, UROLOGY, Adult urology < UROLOGY

For peer review only - http://bmjopen.bmj.com/site/about/guidelines.xhtml

I, the Submitting Author has the right to grant and does grant on behalf of all authors of the Work (as defined in the below author licence), an exclusive licence and/or a non-exclusive licence for contributions from authors who are: i) UK Crown employees; ii) where BMJ has agreed a CC-BY licence shall apply, and/or iii) in accordance with the terms applicable for US Federal Government officers or employees acting as part of their official duties; on a worldwide, perpetual, irrevocable, royalty-free basis to BMJ Publishing Group Ltd ("BMJ") its licensees and where the relevant Journal is co-owned by BMJ to the co-owners of the Journal, to publish the Work in this journal and any other BMJ products and to exploit all rights, as set out in our licence.

The Submitting Author accepts and understands that any supply made under these terms is made by BMJ to the Submitting Author unless you are acting as an employee on behalf of your employer or a postgraduate student of an affiliated institution which is paying any applicable article publishing charge ("APC") for Open Access articles. Where the Submitting Author wishes to make the Work available on an Open Access basis (and intends to pay the relevant APC), the terms of reuse of such Open Access shall be governed by a Creative Commons licence – details of these licences and which Creative Commons licence will apply to this Work are set out in our licence referred to above.

Other than as permitted in any relevant BMJ Author's Self Archiving Policies, I confirm this Work has not been accepted for publication elsewhere, is not being considered for publication elsewhere and does not duplicate material already published. I confirm all authors consent to publication of this Work and authorise the granting of this licence.

For peer review only

Protocol for PRImary care Management of lower Urinary tract Symptoms in men: Protocol for development and validation of a diagnostic and clinical decision support tool (The PriMUS Study).

Title: Protocol for PRImary care Management of lower Urinary tract Symptoms in men: Protocol for development and validation of a diagnostic and clinical decision support tool (The PriMUS Study).

For peer review only

Bethan Pell[1] **(corresponding author)**, Emma Thomas-Jones[1], Alison Bray[2,3], Ridhi Agarwal[4], Haroon Ahmed[5], A Joy Allen[6], Samantha Clarke[7], Jonathan J Deeks[8], Marcus Drake[7], Michael Drinnan[2,3], Calie Dyer[1], Kerenza Hood[1], Natalie Joseph-Williams[5], Lucy Marsh[1], Sarah Milosevic[1], Robert Pickard[9], Tom Schatzberger[10], Yemisi Takwoingi[4], Chris Harding[9], Adrian Edwards[5]

Author Affiliations

[1]Centre for Trials Research, Cardiff University, Heath Park, Cardiff CF14 4YS, UK

[2]Medical Physics, Newcastle Upon Tyne NHS Hospital Trust, Newcastle Freeman Hospital, Freeman Rd, High Heaton, Newcastle upon Tyne NE7 7DN

[3]Translational and Clinical Research Institute, Newcastle University, Newcastle upon Tyne, NE1 7RU

[4]Test Evaluation Research Group, Institute of Applied Health Research, University of Birmingham, Birmingham, B15 2TT

[5]Division of Population Medicine, School of Medicine, Neuadd Meirionnydd, Cardiff University, Cardiff CF14 4YS, UK

[6]National Institute for Health Research (NIHR) Newcastle In Vitro Diagnostics Co-operative, Translational and Clinical Research Institute, Medical School, Newcastle University, Newcastle upon Tyne NE2 4HH

[7]North Bristol NHS Trust, Southmead Hospital, Southmead Road, Westbury-on-Trym, Bristol, BS10 5NB

Protocol for PRImary care Management of lower Urinary tract Symptoms in men: Protocol for development and validation of a diagnostic and clinical decision support tool (The PriMUS Study).

[8]University of Birmingham, Public Health, Epidemiology and Biostatistics, Birmingham, UK

[9]Department of Urology, Newcastle Upon Tyne NHS Hospital Trust, Newcastle Freeman Hospital, Freeman Rd, High Heaton, Newcastle upon Tyne NE7 7DN

[10] Corbridge Health Centre, NHS Northumberland Care Commissioning Group, Newcastle Road, Corbridge, NE45 5LG

Corresponding Author Information

Bethan Pell pellb@cardiff.ac.uk

Address: 7th floor Neuadd Meirionnydd, Heath Park, Cardiff CF14 4YS

Word Count

4407

For peer review only

Protocol for PRImary care Management of lower Urinary tract Symptoms in men: Protocol for development and validation of a diagnostic and clinical decision support tool (The PriMUS Study).

ABSTRACT

Introduction: Lower Urinary Tract Symptoms (LUTS) is a bothersome condition affecting older men that can lead to poor quality of life. General Practitioners (GPs) currently have no easily available assessment tools to help effectively diagnose causes of LUTS and aid discussion of treatment with patients. Men are frequently referred to urology specialists who often recommend treatments that could have been initiated in Primary Care. GP access to simple, accurate tests and clinician decision tools are needed to facilitate accurate and effective patient management of LUTS in primary care.

Methods and analysis: PriMUS is a prospective diagnostic accuracy study based in primary care. The study will determine which of a number of index tests used in combination, best predict three urodynamic observations in men who present to their GP with LUTS. These are detrusor overactivity, bladder outlet obstruction, and/or detrusor underactivity. Two cohorts of participants, one for development of the prototype diagnostic tool, and one for validation, will undergo a series of simple index tests and the invasive reference standard (invasive urodynamics). We will develop and validate three diagnostic prediction models based on each condition, and then combine them with management recommendations to form a clinical decision support tool.

Ethics and dissemination: Ethics approval is from the Wales Research Ethics Committee 6. Findings will be disseminated through peer-reviewed journals and conferences; results will be of interest to professional and patient stakeholders.

Study registration: ISRCTN10327305

Strengths and limitations of this study

- Prospective, multicentre study in an appropriate population in primary care.
- The index tests are tests that can be done routinely in primary care or at home by patients.
- The diagnostic models developed will be validated in a separate cohort of men from the same population.
- The assumed prevalence of the three target conditions may be different in practice.
- Some test results may be missing or difficult to obtain.

Protocol version: 7.0

Keywords: primary care, urology, adult urology

For peer review only

Protocol for PRImary care Management of lower Urinary tract Symptoms in men: Protocol for development and validation of a diagnostic and clinical decision support tool (The PriMUS Study).

INTRODUCTION

Lower urinary tract symptoms (LUTS), such as frequent urination, a slow stream, and having to wake in the night to urinate, affect a significant proportion of older men and can lead to poor quality of life. Three common causes of LUTS are: instability of the bladder muscle (detrusor overactivity, DO), benign enlargement of the prostate gland causing bladder outlet obstruction (BOO), and weakness of the bladder muscle (detrusor underactivity, DU). These may be present individually or in combination.

The reference standard test for investigation of LUTS, and thus diagnosis of DO, BOO and DU, is invasive urodynamics, which takes place in secondary care. Invasive urodynamics will be conducted rather than video urodynamics, which is in line with most contemporary national and international guidelines, and is sufficient to diagnose DO, BOO and DU, to which most non-complicated adult male LUTS can be attributed. It involves insertion of catheters into the patient's bladder and rectum so that the behaviour of the bladder and outlet can be examined during filling and voiding. Owing to availability, complexity and cost, management decisions for men with LUTS are usually based on results from a combination of non- and minimally-invasive investigations instead. These include digital rectal examination (DRE) to assess prostate size, symptoms questionnaires, uroflowmetry, and measurement of post void residual.

NICE Guidelines suggest that many men referred to specialist care with LUTS are eventually managed conservatively, and so could have remained within primary care. Male LUTS

For peer review only - http://bmjopen.bmj.com/site/about/guidelines.xhtml

account for around four presentations per month in an average-sized General Practitioner (GP) practice. This rate of presentation, although high enough to represent a large burden on the National Health Service (NHS), makes it difficult for GPs to gain sufficient expertise to be confident about diagnosis and management. Further, GPs do not have access to simple tools giving an indication of the most likely cause of symptoms to guide treatment and management. Making such a tool available should improve treatment efficacy, standardise treatment, reduce unnecessary referrals, expedite referral of those requiring specialist care, and thus improve cost-effectiveness of NHS care.

This led to the National Institute for Health Research (NIHR) releasing a 2015 health technology assessment (HTA) commissioned call (HTA number 15/40) seeking the delivery of: *The development of a decision aid to help inform the choice of treatment or need for specialist referral for men presenting with lower urinary tract symptoms in primary care*. Our team was successful in obtaining this funding and here we describe the protocol for our study: *Primary care management of lower urinary tract symptoms in men: Development and validation of a diagnostic and clinical decision support tool (The PriMUS Study)*.

Aims, Objectives and Outcome measures

The PriMUS study aims to develop three diagnostic prediction models based on the results of simple clinical tests that can provide a useful prediction of urodynamic observations in men with LUTS. We will assess the diagnostic accuracy of these models, which will be implemented in software, along with management recommendation algorithms to form a clinical decision support tool for use in UK primary care. Our primary and secondary objectives and measures are outlined below.

Primary Objectives

- Develop a statistical model to predict the likelihood of three urological conditions (bladder outlet obstruction, detrusor overactivity, detrusor underactivity) based on a series of non-invasive index tests, with invasive urodynamics as the reference standard.
- Estimate the diagnostic accuracy of the above statistical model in an independent validation cohort.

Secondary Objectives

- Develop a series of patient management recommendations and thresholds for clinically useful diagnostic prediction by expert consensus and with reference to current clinical guidelines that map to the diagnoses predicted by the statistical model.

For peer review only

Protocol for PRImary care Management of lower Urinary tract Symptoms in men: Protocol for development and validation of a diagnostic and clinical decision support tool (The PriMUS Study).

- Combine the statistical model and management recommendations into an online tool that will form the prototype clinical decision support tool.
- Complete a qualitative study to explore the feasibility of introducing the clinical decision support tool into primary care including potential acceptability to primary care staff and patients.
- Collect NHS costs involved in delivering the new pathway and compare with cost of standard pathway calculated from NHS and other sources.

Primary outcome

Sensitivity and specificity of the diagnostic models for detecting detrusor underactivity, bladder outlet obstruction and detrusor over activity will be determined. The three conditions will be coded as binary outcomes (present/absent).

Secondary outcomes

- A patient management algorithm to guide initial treatment for men with LUTS.
- A prototype online clinical decision support tool for use in primary care.
- Qualitative summary of patients' and clinicians' views on the use of a LUTS clinical decision support tool in the primary care setting.
- Costs / savings of implementation of the primary care LUTS clinical decision support tool both from a population and individual patient perspective.

METHODS AND ANALYSIS

Study design

This is a prospective, diagnostic accuracy study involving the development and validation of a diagnostic tool. An internal pilot will assess primary care recruitment, acceptability of the reference test (invasive urodynamics) and data collection. Two cohorts of participants, one for development of the prototype diagnostic tool, and one for validation, will undergo a series of index tests (see Table 1) and the invasive reference standard (urodynamics) in approximately 90 GP practices across Newcastle Upon Tyne, Wales and Bristol (a list of study sites can be found on ISRCTN). There will also be qualitative data collection to explore acceptability of the urodynamics test, develop management recommendations for the tool and for user-testing of the prototype (see Table 2).

Participants

Adult men who consult their GP with one or more LUTS in UK primary care settings.

Inclusion criteria

- Men aged 16 years and over.
- Men who present to their GP with a complaint of one or more bothersome lower urinary tract symptoms (this includes men on current treatment, but who are still symptomatic).
- Men able and willing to give informed consent for participation in study.

For peer review only - http://bmjopen.bmj.com/site/about/guidelines.xhtml

- Men able and willing to undergo all index tests and reference test, and complete study documentation.

Exclusion criteria

- Men with neurological disease or injury affecting lower urinary tract function.
- Men with LUTS considered secondary to current or past invasive treatment or radiotherapy for pelvic disease.
- Men with contraindications to urodynamics such as heart valve or joint replacement surgery within the last three months, or immunocompromised/immunosuppressed men.
- Men with indwelling urinary catheters or who carry out intermittent self-catheterisation.
- Men whose initial assessment suggests that clinical findings are suggestive of:
  - Prostate or bladder cancer according to standard NHS cancer pathways. If later deemed unlikely, they become eligible for study participation.
  - Recurrent or persistent symptomatic urinary tract infection (UTI). If UTI is successfully treated but LUTS remain, they become eligible for study participation.
  - Urinary retention, for example palpable bladder after voiding.
- Men unable to consent in English or Welsh where a suitable translator is not available.

Test Selection

Test selection, including the reference standard, was informed by a systematic review included in the relevant NICE guideline CG97[1] updated with a study-specific unpublished selective review by our group in 2015, the judgement of the expert clinical members of the study team, and the stipulations of the funding commissioning brief. All participants undergo all tests, which are a combination of:

- Tests carried out for eligibility assessment prior to enrolment, as described above.
- Tests carried out at participant visits to a primary or secondary care location for the purpose of the study following enrolment.
- Tests carried out by the participant at home following enrolment.

Index Tests

For peer review only - http://bmjopen.bmj.com/site/about/guidelines.xhtml

Protocol for PRImary care Management of lower Urinary tract Symptoms in men: Protocol for development and validation of a diagnostic and clinical decision support tool (The PriMUS Study).

Twelve potential parameters will be considered for the three logistic regression models. The investigations that provide these parameters are described in Table 1 below.

Table 1. Index Tests and Input parameters that will be tested for use in the three logistic regression models.

Test	*Result*	*Input parameters that will be tested for use in the three logistic regression models. (result or unit)*
Relevant demographics	Age in years	Age (years)
Physical examination of abdomen	Bladder palpable/not palpable	N/A
Digital rectal examination	Prostate mild/moderate/severe enlargement<br>Further assessment for prostate cancer required/not required	Prostate size (enlarged/not enlarged)
Prostate specific antigen	PSA value – established thresholds for further assessment for prostate cancer (typically > 3 ng/ml) or benign enlargement (typically ≥ 1.5 ng/ml)<br>For clinical decision support tool: continuous variable in ng/mL	Prostate specific antigen (ng/ml)
International Consultation on Incontinence Questionnaire Male Lower Urinary Tract Symptoms Short Form (ICIQ M-LUTS)	Total score (0-52); Voiding symptom score (0-20), storage symptom score (0-24). Individual symptom bother scores scored separately from symptom severity scores (0-130).	Storage/incontinence symptoms sub-score<br>Voiding symptoms sub-score
International Prostate Symptom Score (IPSS) questionnaires.	Total score (0-35)	Storage/incontinence symptoms sub-score<br>Voiding symptoms sub-score
Bladder Diary	Waking (day) time frequency, sleeping (night) time frequency, 24 hour voided volume, daytime voided volume, nocturnal voided volume, average volume voided each void, total urgency scores	Mean urgency score<br>Mean 24-hour fluid intake (ml)
Uroflowmetry (Flowtaker)	Maximum flow rate, voided volume against normal age-adjusted range. Single value in ml/s	Median maximum flow rate (ml/s)<br>Median voided volume (ml)<br>Mean 24-hour frequency<br>Mean nocturia
Post void residual	Residual volume against normal age-adjusted range. Single value in ml	Post void residual volume (ml)

Reference Standard

For peer review only - http://bmjopen.bmj.com/site/about/guidelines.xhtml

Our reference standard is invasive urodynamics, a test routinely carried out in a specialist care setting for the investigation of LUTS. For this study, it will be performed using portable equipment (Goby, Laborie, Mississauga, Canada) in either a primary or secondary care location, by specially-trained urodynamic nurses, according to International Continence Society (ICS) standards.[2] Safety information is covered in the *Safety* section below.

Study Procedures

Data Collection

GPs, primary care nurses or an appropriately trained delegate will undertake the data collection relating to all the index tests. Specialist trained urodynamic nurses will undertake the data collection for the reference test.

Data Management

All data collection will be by electronic data capture using a bespoke database developed by the Cardiff University Centre for Trials Research Clinical Trials Unit (CTR), and paper copies of all case report forms (CRFs) will be available.

Identification and Screening

All men will be identified either opportunistically during a GP consultation, or by regular, pre-defined primary care database searches. They must undergo three screening tests prior to enrolment into the study; a physical examination of the abdomen (palpable bladder check), DRE and Prostate Specific Antigen (PSA) test. The latter two test results are accepted if they have undergone these investigations within the last six months.

Informed Consent

Informed consent will be obtained in the first study visit (Study Visit Part A) prior to any study procedures by those suitably trained and on the delegation log. Eligible patients will be given time to consider before being asked to sign the consent form. Once consented, participants will be allocated a unique study number (participant ID).

Separate informed consent will be taken for participation in the qualitative data collection.

Withdrawal

Patients will be notified that they can withdraw consent for their participation in the study at any time during the study period.

Study Visit Part A

Once informed consent is obtained, the remaining index tests will be collected. This includes a baseline assessment (collecting demographic information, relevant medication and medical history) and two self-reported questionnaires; IPSS and ICIQ. Participants will be

For peer review only

Protocol for PRImary care Management of lower Urinary tract Symptoms in men: Protocol for development and validation of a diagnostic and clinical decision support tool (The PriMUS Study).

given the bladder diary to complete for three days at home and instructed to bring this to their invasive urodynamic visit (Study Visit Part B).

Study Visit Part B - Reference standard

On arrival, the patient will be asked to pass urine into a flowmeter in private, after which a measurement of post void residual ultrasound (one of the index tests; see below) will be made. A dual lumen catheter (one channel to fill the bladder, and the other to measure intravesical pressure, P_{ves}) will be inserted into the bladder via the urethra, and a single lumen catheter inserted into the rectum to measure abdominal pressure (P_{abd}). Detrusor pressure (P_{det}), generated by the bladder muscle itself, is calculated by subtracting P_{abd} from P_{ves}.

Filling Phase

The patient will be asked to bring their completed bladder diary (one of the index tests; see above) to their urodynamics appointment, providing the urodynamic nurse with an indication of their maximum bladder capacity. The patient's bladder will be filled with sterile saline at a maximum rate of 50 ml/min. They will be asked to report the first sensation of bladder filling, followed by the point at which they feel the normal desire to void, and finally the strong desire to void. At this point bladder filling will be stopped and provocation, in the form of running taps and asking the patient to cough, will be performed.

Voiding Phase

Following provocation, the patient will be given permission to void, marking the start of the voiding phase. Voided volume (V_{void}) and flow rate (Q) will be measured as they pass urine into the flowmeter.

If either the filled or voided volumes are below 150 ml, the filling and voiding phases will be repeated once more using a maximum filling rate of 20 ml/min.

Diagnostic Definitions

For peer review only - http://bmjopen.bmj.com/site/about/guidelines.xhtml

Protocol for PRImary care Management of lower Urinary tract Symptoms in men: Protocol for development and validation of a diagnostic and clinical decision support tool (The PriMUS Study).

Definition of our three target conditions will be based upon the following parameters measured during invasive urodynamics and subsequently read from a graphical representation of the test:

1. Maximum detrusor contraction pressure during the filling phase.
2. Maximum flow rate during the voiding phase (Q_{max}).
3. Detrusor pressure at the point of maximum flow rate ($P_{detQmax}$).

If there are no detrusor contractions during filling, DO is not present. If there are any contractions (contraction pressure>0), DO is present.

Diagnosis of BOO is based upon the bladder outlet obstruction index (BOOI), defined as PdetQmax-2*Qmax. BOO is present if BOOI>40, and absent if BOOI≤40.

Diagnosis of DU is based upon the bladder contractility index (BCI), defined as PdetQmax+5*Qmax. DU is present if BCI<100, and absent if BCI≥100.

Debrief process and monitoring process

The urodynamic nurses will debrief the patient following the urodynamic procedure providing them with a post urodynamics leaflet and safety card. The urodynamic nurse will also instruct the patient that they will receive a 3 Day Follow Up Phone Call, to monitor for any related adverse events.

3 Day Follow Up Phone Call

The urodynamic nurses will contact the patient 3 days (+/- flexibility if phone call falls on weekend) after their urodynamic procedure to monitor for any adverse events and Serious Adverse Events (SAEs). Any SAEs are subsequently recorded by the urodynamic nurses and processed centrally by CTR. This process is outlined in the Safety Section (see below).

Review Process

Invasive urodynamics is a complex investigation and interpretation can be challenging. Further, because standard practice involves interaction between the reference and some index tests as described earlier, their primary interpretation in this study is not blinded.

For peer review only

Protocol for PRImary care Management of lower Urinary tract Symptoms in men: Protocol for development and validation of a diagnostic and clinical decision support tool (The PriMUS Study).

Therefore, a review process will be implemented to ensure the integrity of the reference standard. All studies will be second-read by a blinded reviewer to extract the three parameters above. If any of the resulting diagnoses differ between the nurse and this reviewer, the case will go to a second non-blinded reviewer who makes the final decision.

Uroflowmetry* (Flowtaker)

The patient is provided with the Flowtaker at the end of their invasive urodynamic visit. They will be provided with an information sheet on how to use this and instructed to not start this until given the greenlight to do so during their 3 Day Follow Up Phone Call. The Flowmeter will be given with a pre-paid envelope for the patient to post back to their urodynamic nurse for data upload.

GP Summary Report

Once the reference and index tests have taken place, results are compiled into a report which is provided to GPs, along with a summary of relevant NICE-recommended managements,[1] to help inform management of the patient.

6 Month Follow Up

A review of the patient's medical notes will take place 6 months after the patient's treatment and management decision with the GP. This will include any changes to treatment, or management and whether they have since been referred to secondary care. Figure 1 depicts a flow diagram of the patient pathway.

[INSERT FIGURE 1 HERE]

Safety and Pharmacovigilance

Invasive urodynamics has the potential to cause adverse events. A medical doctor will be required on site whilst the test is taking place. Due to a 5% risk of a urinary tract infection,[3] the urodynamic nurse will also provide the patient with a post-urodynamics debrief sheet following the test, informing them on the importance of drinking plenty of water for 24 hours following the test, how to identify signs of an infection, and to seek medical care if they suspect they have one.

For peer review only

Protocol for PRImary care Management of lower Urinary tract Symptoms in men: Protocol for development and validation of a diagnostic and clinical decision support tool (The PriMUS Study).

Adverse events will be captured by the urodynamic nurses, either during Study Visit Part B, or during the 3 day follow up phone call. For serious adverse events (SAEs), an assessment of causality between the event and the study intervention, and the expectedness of the event, will be carried out by the carried out by the PI, or delegated urodynamic nurse, and then independently by a clinical reviewer. If the clinical reviewer classes the event as *probably* or *definitely* caused by the intervention, it will be classified as a serious adverse reaction.

Sample Size

Sample size calculations were carried out separately for the model development and validation cohorts. For both we used estimated prevalences for DO, BOO and DU of 57%, 31% and 16% respectively based on previous literature[4,5] and clinical expertise.

Development Cohort

The sample size for developing our predictive models was based on a rule of thumb suggesting that five events per variable are required.[6] We chose a sample size of 350 to allow at least 11 variables in each model. This was driven by our lowest estimated prevalence of 16% for DU, giving 56 events (DU diagnoses).

Validation Cohort

The sample size for the validation cohort was chosen to ensure that estimates of test accuracy are made with adequate precision. We deem sensitivity and specificity of 75% to be the minimum clinically useful performance. We chose a sample size of 325, giving estimates of sensitivity of 75% to within 8%, 10% and 14% for DO, BOO and DU respectively, based on ‘positive’ samples of 185, 101 and 52, and estimates of specificity of 75% to within 8%, 7% and 6%, based on ‘negative’ samples of 140, 224 and 273. Better sensitivity and specificity will give narrower confidence intervals.

Attrition

In order to allow an attrition rate of 20-25%, the resulting total of 675 was increased to give a final sample size of 880.

For peer review only

Protocol for PRImary care Management of lower Urinary tract Symptoms in men: Protocol for development and validation of a diagnostic and clinical decision support tool (The PriMUS Study).

STATISTICAL ANALYSIS

Model development will be performed using results from the first 350 datasets, and external model validation performed using the subsequent 325 datasets.

Model Development

Candidate predictor variables will be selected from those listed in Table 1. Their selection has been informed by subject knowledge using literature review and expert judgement. As predictor distributions should be wide to facilitate reliable predictions, we will explore the distribution of each predictor prior to selection. Relationships between predictors will also be investigated; where indicated we will group related variables into a composite variable or exclude if highly correlated with other variables. Candidate predictors will not be selected based on univariable analyses; this practice is discouraged because predictors that may be important in a multivariable model can be missed and may also lead to overoptimistic models. Therefore, all selected candidate predictor variables will be included in the multivariable logistic regression models without evaluations of association between outcome and predictor and assessment of statistical significance. To gain maximum diagnostic information, continuous variables will not be categorised. We will allow for non-linearity by using a multivariable fractional polynomial (MFP) approach to identify appropriate transformations. This may lead to the inclusion of non-linear terms in the models thus increasing the number of variables in the models. Using multiply imputed data and Rubin's rule, we will develop each model using backward elimination with a p value of 0.157 to select predictors for inclusion in each model. We chose this p value because it is known to be a good proxy for the Akaike information criterion (AIC) approach. If the repeated use of Rubin's rule is computationally challenging, we will use the approximation to Rubin's rule recommended by Wood et al (2008).[7]

Model Validation

The predictive performance of each model will be assessed in terms of discrimination, that is the ability to distinguish between those who do or do not have a particular diagnosis, and calibration meaning agreement between predicted and observed probabilities. Discriminative ability will be assessed using the c-index and its 95% confidence intervals. For a logistic model, this is equivalent to the area under the ROC curve (AUC). Calibration will be evaluated in two ways. Calibration plots of average observed probability against predicted probability will be used to visually assess calibration. Within each quintile or decile of predicted probability (depending on the distribution of data), the average predicted probability will be compared with the corresponding observed proportions. We will also quantify calibration by estimating the calibration slope of the prognostic index (linear predictor) using logistic regression with the linear predictor as the covariate.

Protocol for PRImary care Management of lower Urinary tract Symptoms in men: Protocol for development and validation of a diagnostic and clinical decision support tool (The PriMUS Study).

The apparent c-index and calibration slope will be estimated for each model. Bootstrapping will be used for internal validation to assess model overfitting and optimism. For each model, we will obtain 100 bootstrap samples from each imputed dataset and repeat the variable selection process. The optimism is the difference between the c-index from the bootstrap sample and that from the original imputed dataset. The average optimism will be determined across bootstrap samples and imputed datasets, and the optimism-adjusted c-index will be calculated by subtracting the average optimism from the apparent c-index of the original model. Similarly, we will obtain the optimism adjusted calibration slope. The optimism-adjusted calibration slope will be used as the uniform shrinkage factor to correct a model.

We will externally validate the models and calculate performance statistics (c-index and calibration slope) using the validation cohort. The value for the calibration slope should ideally be one signifying perfect agreement between the predicted probabilities and the observed probabilities. A calibration slope < 1 indicates that a model over-predicts while a calibration slope > 1 indicates under prediction. From the qualitative research we will ascertain distributions of probability (risk) thresholds for clinical usefulness of the prediction in guiding treatment of each condition. The sensitivity and specificity (and their corresponding 95% confidence intervals), will be calculated for these risk thresholds and plotted on an ROC plot for each model.

Missing Data

Patterns of missing data will be investigated to infer the ease with which each parameter can be obtained in practice. In the event of missing data, multiple imputation by chained equations[8] will be used to impute missing values in order to avoid bias and make best use of the data.

SECONDARY SUB-STUDIES

Table 2. Secondary Sub-Studies

Qualitative Studies	Details
Patient and Clinician Acceptability Interviews – Internal Pilot Phase	During the pilot phase we will conduct semi-structured qualitative interviews (n=30-40) with patients (consenting and declining entry to the main study) and participating clinicians to assess the acceptability

For peer review only

Protocol for PRImary care Management of lower Urinary tract Symptoms in men: Protocol for development and validation of a diagnostic and clinical decision support tool (The PriMUS Study).

	of the urodynamic procedure and PriMUS Study, as part of our progression criteria. Interview schedules will be developed in discussion with clinician and patient representatives of the Study Management Group (SMG). Interview guides will broadly explore: practicality and acceptability of conducting urodynamic investigations (and experiences of patient participants); reactions to and experiences of the study processes (including barriers/facilitators). An iterative approach will be taken, so that schedules can be refined to further explore unanticipated themes that arise during data collection. Interview transcripts will be entered into NVivo qualitative analysis software and analysed using Framework Analysis (using key topic areas as the framework).[9] Data will be used to inform strategies that will maximise recruitment and retention.
Development of Management Recommendations	Algorithms are required to link outputs from the statistical models, which will be likelihoods of each target condition, with patient management recommendations to form the clinical decision support tool. The starting point will be recommendations from the relevant NICE clinical guideline. Qualitative work with urologists will support the development of these management recommendations, through posing a range of clinical case scenarios to urologists using interview and questionnaire methodologies (n=15-20). Urologists will be asked to how they would manage these scenarios, with a focus on thresholds for treatment and strategies for multiple diagnoses.
Tool Feasibility Assessment	The aim of the user-testing phase will be to build on the interviews conducted as part of the pilot phase evaluation to assess GPs' attitudes and reactions to the prototype clinical decision support tool. GPs (n=10-12) will be sent the tool prior to the semi-structured telephone interview and asked to use it. The interview schedule will explore the following: ease of use, content, design, and perceived acceptability and feasibility of using the tool in routine primary care settings (allowing succinct exploration of the prototype tool). Interview transcripts will be entered into NVivo qualitative analysis software and analysed using Framework Analysis (using key topic areas as the framework).[9] Feedback will be used to improve and refine the tool.

For peer review only - http://bmjopen.bmj.com/site/about/guidelines.xhtml

Protocol for PRImary care Management of lower Urinary tract Symptoms in men: Protocol for development and validation of a diagnostic and clinical decision support tool (The PriMUS Study).

STUDY MANAGEMENT

The study is sponsored by Cardiff University, coordinated by CTR and co-led by The Newcastle upon Tyne Hospitals NHS Foundation Trust (NuTH). The other partner organisations will be Birmingham University, University of Bristol, and North of England Commissioning Support.

Study management Group

The Study Management Group (SMG) will meet monthly throughout the course of the study and will include the Chief Investigators, co-applicants, collaborators, study manager, data manager and administrator. Two patient representatives will also attend and contribute to the conception, design and management of the study, as well as patient-facing materials. SMG members will be required to sign up to the remit and conditions as set out in the SMG Charter.

Study Steering Committee

An independent Study Steering Committee (SSC) consisting of an independent chairperson, two independent members and two patient representatives will provide oversight of The PriMUS Study. Instead of a separate Independent Data Monitoring Committee (IDMC), the SSC will also provide oversight of all matters relating to patient safety and data quality. Members will be required to sign up to the remit and conditions as set out in the SSC Charters and will meet annually.

ETHICS AND DISSEMINATION

Research approvals

The Wales REC 6 has approved the study (17/WA/0155) on the 20th June 2017 and subsequent R&D Approval for Wales on 21st August 2017 and HRA Approval on 23rd August 2017.

All study participants will give informed consent before taking part (see earlier).

The following substantial amendments were made to the trial and were communicated to all trial sites: Substantial Amendment 1 (3rd October 2017); Substantial Amendment 2 (10th January 2018); Substantial Amendment 3 (20th April 2018); Substantial Amendment 4 (26th February 2019); Substantial Amendment 5 (6th June 2019); Substantial Amendment 6 (6th September 2019)

The study has the following registration: **ISRCTN10327305**

Dissemination plan

Following completion of the study, a final report will be prepared for the National Institute of Health Research (NIHR) Journal series. A paper describing the primary results will be submitted to a high impact, international, peer-reviewed journal. Qualitative studies and sub-studies will also be submitted for publication. We will present our findings at national and international scientific meetings.

For peer review only - http://bmjopen.bmj.com/site/about/guidelines.xhtml

Protocol for PRImary care Management of lower Urinary tract Symptoms in men: Protocol for development and validation of a diagnostic and clinical decision support tool (The PriMUS Study).

With the assistance of our collaborators and lay representatives we will disseminate the study findings to a wide NHS and general audience and promote uptake of outputs into clinical care. This will include presentations at meetings and written executive summaries for key stakeholder groups such as Primary Care Trusts, Secondary Care Trusts, Health Boards, Royal Colleges, Medical Schools, and relevant patient groups.

All publications and presentations relating to the study will be authorised by the SMG in accordance with the study's publication policy.

Competing Interests Statement

One of the index tests, Flowtaker, was developed by a team from NuTH and Newcastle University, including two individuals who are grant co-applicants, members of the study management team and co-authors (Authors 3 and 10). In 2014 the device was licensed to MMS (Enschede, The Netherlands) and royalties from the sale of the device were paid to NuTH (not to the individuals). MMS was subsequently acquired by Laborie who removed Flowtaker from the market in January 2018.

Author Contributions

AE and CH are Co-Chief Investigators of this study. AE and CH led the development of the research question, study design, obtaining the funding, and implementation of the study protocol, along with HA, JA, AB, MD, JD, MD, KH, NJW, RP, TS, YT and ETJ. BP is the Study Manager and ETJ the Senior Study Manager who coordinated the operational delivery of the study protocol and recruitment. SC provides research nurse insight and support. NJW and SM are the qualitative researchers. YT and RA are the study statisticians. CD and LM are the data managers. All authors listed provided critical review and final approval of the manuscript.

Funding

This study is funded by the NIHR (Health Technology Assessment programme), funder reference 15-40-05.

Disclaimer

For peer review only - http://bmjopen.bmj.com/site/about/guidelines.xhtml

Protocol for PRImary care Management of lower Urinary tract Symptoms in men: Protocol for development and validation of a diagnostic and clinical decision support tool (The PriMUS Study).

The views expressed are those of the authors and not necessarily those of the NHS, the NIHR or the Department of Health and Social Care. Neither the Sponsor nor the Funder had any role on the study design; collection, management, analysis and interpretation of data; writing of this manuscript or in the decision to submit this manuscript for publication.

Sponsor

Cardiff University, Research and Innovation Services Department, Contracts Team, Cardiff University, 30-36 Newport Road, Cardiff, CF24 0DE. Contact person: Ms Helen Falconer; FalconerHE@cardiff.ac.uk. Sponsor reference: SPON 1553-16

Acknowledgements

Professor Robert Pickard † for development of the research question, study design, obtaining the funding, and implementation of the study protocol. The authors would like to thank the research nurses for their support during the study Debra Barnett, Laura Bevan, Jane Davies, Alison Edwards, Gareth Kennard-Holden, Lisa Mellish, Joanne Sullivan, Joanne Thompson.

The Centre for Trials Research receives funding from Health and Care Research Wales and Cancer Research UK.

JA is supported by the National Institute for Health Research (NIHR) Newcastle In Vitro Diagnostics Co-operative. The views expressed are those of the authors and not necessarily those of the NIHR or the Department of Health and Social Care.

The authors would also like to acknowledge the contribution of the Trial Steering Committee members, namely Professor Tom Fahey, Professor Rafael Perea, Dr Gail Hayward, Dr Ian Pearce and Mr Alan Pryce.

Patient and Public Involvement

The authors would like to acknowledge the contribution of Malcom Gristwood and Ray White as the public and patient involvement representatives on the Study Management Group.

Consent for publication

Not Applicable

Availability of data and material

† Deceased 24 July 2018

For peer review only

Protocol for PRImary care Management of lower Urinary tract Symptoms in men: Protocol for development and validation of a diagnostic and clinical decision support tool (The PriMUS Study).

Not Applicable

REFERENCES

1. National Institute for Health and Clinical Excellence (NICE). *The management of lower urinary tract symptoms in men: Clinical guideline 97.* May 2010.

2. Drake M, Doumouchtsis SK, Hashim H, et al. Fundamentals of urodynamic practice, based on International Continence Society good urodynamic practices recommendations. *Neurourol Urodyn.* 2018;37(S6):S50-S60. doi: 10.1002/nau.23773.

3. Foon R, Toozs-Hobson P, Latthe P. Prophylactic antibiotics to reduce the risk of urinary tract infections after urodynamic studies. *Cochrane Database Syst Rev* 2012;10. Art. No.: CD008224. DOI: 10.1002/14651858.CD008224.pub2.

4. Kuo HC. Videourodynamic analysis of pathophysiology of men with both storage and voiding lower urinary tract symptoms. *Urology.* 2007;70:272–276.

5. van Doorn B, Blanker MH, Kok ET, et al. Prevalence, incidence, and resolution of nocturnal polyuria in a longitudinal community-based study in older men: The Krimpen study. *Eur Urol.* 2013;63:542-554.

6. Vittinghoff E, McCulloch CE. Relaxing the rule of ten events per variable in logistic and Cox regression. *Am J Epidemiol.* 2007;165(6):710-8.

7. Wood AM, White IR, Royston P. How should variable selection be performed with multiply imputed data? *Stat Med.* 2008;27:3227–3246.

8. van Buuren S, Boshuizen HC, Knook DL. Multiple imputation of missing blood pressure covariates in survival analysis. *Stat Med.* 1999;18(6):681-94.

9. Gale, NK, Heath, G, Cameron, E, et al. Using the framework method for the analysis of qualitative data in multi-disciplinary health research. *BMC Med Res Methodol* 2013;13:117. https://doi.org/10.1186/1471-2288-13-117.

Figure 1 Legend – Flow Diagram depicting the Patient Pathway in The PriMUS Study.

For peer review only

Screening and Confirmation of Eligibility

The GP will confirm eligibility to enter the study. The test results needed to confirm eligibility are: digital rectal examination, physical examination and prostate specific antigen blood test.

↓

Study Visit (Part A)

- Consent *(If not already taken in GP Visit).*
- Collection/Provision of Bladder Diary.
- Two self-reported questionnaires.
- Questions regarding medical history, relevant medical and demographics.

↓

Study Visit (Part B)

- Urodynamics reference test.
- Uroflowmetry (Flowtaker).

↓

Follow Up Phone Call

The nurse who conducted the urodynamics test will call the patient 3 days after the test to monitor any adverse events.

If the nurse then feels there are no ongoing side effects from the urodynamics, the patient will start using the Flowtaker.

↓

GP Summary Report

Once all the results are available, they will be fed back to the GP. An appointment will be arranged with the patient for further treatment and management of LUTS.

ew only - http://bmjopen.bmj.com/site/about/guid

↓

6 Month Note Review

Review of the patient's medical notes will take place 6 months after the patient's treatment and management decision with the GP.

TRIPOD Checklist: Prediction Model Development and Validation

Section/Topic	Item		Checklist Item	Page
**Title and abstract**				
Title	1	D;V	Identify the study as developing and/or validating a multivariable prediction model, the target population, and the outcome to be predicted.	1
Abstract	2	D;V	Provide a summary of objectives, study design, setting, participants, sample size, predictors, outcome, statistical analysis, results, and conclusions.	1
**Introduction**				
Background and objectives	3a	D;V	Explain the medical context (including whether diagnostic or prognostic) and rationale for developing or validating the multivariable prediction model, including references to existing models.	2
	3b	D;V	Specify the objectives, including whether the study describes the development or validation of the model or both.	2-3
**Methods**				
Source of data	4a	D;V	Describe the study design or source of data (e.g., randomized trial, cohort, or registry data), separately for the development and validation data sets, if applicable.	3
	4b	D;V	Specify the key study dates, including start of accrual; end of accrual; and, if applicable, end of follow-up.	N/A
Participants	5a	D;V	Specify key elements of the study setting (e.g., primary care, secondary care, general population) including number and location of centres.	3
	5b	D;V	Describe eligibility criteria for participants.	3-4
	5c	D;V	Give details of treatments received, if relevant.	4-5
Outcome	6a	D;V	Clearly define the outcome that is predicted by the prediction model, including how and when assessed.	3
	6b	D;V	Report any actions to blind assessment of the outcome to be predicted.	
Predictors	7a	D;V	Clearly define all predictors used in developing or validating the multivariable prediction model, including how and when they were measured.	4
	7b	D;V	Report any actions to blind assessment of predictors for the outcome and other predictors.	4
Sample size	8	D;V	Explain how the study size was arrived at.	8
Missing data	9	D;V	Describe how missing data were handled (e.g., complete-case analysis, single imputation, multiple imputation) with details of any imputation method.	9
Statistical analysis methods	10a	D	Describe how predictors were handled in the analyses.	8-9
	10b	D	Specify type of model, all model-building procedures (including any predictor selection), and method for internal validation.	8-9
	10c	V	For validation, describe how the predictions were calculated.	8-9
	10d	D;V	Specify all measures used to assess model performance and, if relevant, to compare multiple models.	8-9
	10e	V	Describe any model updating (e.g., recalibration) arising from the validation, if done.	N/A
Risk groups	11	D;V	Provide details on how risk groups were created, if done.	N/A
Development vs. validation	12	V	For validation, identify any differences from the development data in setting, eligibility criteria, outcome, and predictors.	8-9
**Results**				
Participants	13a	D;V	Describe the flow of participants through the study, including the number of participants with and without the outcome and, if applicable, a summary of the follow-up time. A diagram may be helpful.	6-7
	13b	D;V	Describe the characteristics of the participants (basic demographics, clinical features, available predictors), including the number of participants with missing data for predictors and outcome.	6
	13c	V	For validation, show a comparison with the development data of the distribution of important variables (demographics, predictors and outcome).	N/A
Model development	14a	D	Specify the number of participants and outcome events in each analysis.	8
	14b	D	If done, report the unadjusted association between each candidate predictor and outcome.	N/A
Model specification	15a	D	Present the full prediction model to allow predictions for individuals (i.e., all regression coefficients, and model intercept or baseline survival at a given time point).	N/A
	15b	D	Explain how to the use the prediction model.	N/A
Model performance	16	D;V	Report performance measures (with CIs) for the prediction model.	N/A
Model-updating	17	V	If done, report the results from any model updating (i.e., model specification, model performance).	N/A
**Discussion**				
Limitations	18	D;V	Discuss any limitations of the study (such as nonrepresentative sample, few events per predictor, missing data).	N/A
Interpretation	19a	V	For validation, discuss the results with reference to performance in the development data, and any other validation data.	N/A
	19b	D;V	Give an overall interpretation of the results, considering objectives, limitations, results from similar studies, and other relevant evidence.	N/A
Implications	20	D;V	Discuss the potential clinical use of the model and implications for future research.	N/A
**Other information**				
Supplementary information	21	D;V	Provide information about the availability of supplementary resources, such as study protocol, Web calculator, and data sets.	N/A
Funding	22	D;V	Give the source of funding and the role of the funders for the present study.	11

*Items relevant only to the development of a prediction model are denoted by D, items relating solely to a validation of a prediction model are denoted by V, and items relating to both are denoted D;V. We recommend using the TRIPOD Checklist in conjunction with the TRIPOD Explanation and Elaboration document.

For peer review only
